# Promoting metabolic inefficiency for metabolic disease

**DOI:** 10.1016/j.isci.2023.107843

**Published:** 2023-09-06

**Authors:** Lawrence Kazak

**Affiliations:** 1 Rosalind & Morris Goodman Cancer Institute, McGill University, Montreal, QC H3A 1A3, Canada; 2 Department of Biochemistry, McGill University, Montreal, QC H3G 1Y6, Canada

**Keywords:** Human metabolism

## Abstract

Recent advances in pharmacotherapies that promote appetite suppression have shown remarkable weight loss. Therapies targeting energy expenditure lag behind, and as such none have yet been identified to be safe and efficacious for sustaining negative energy balance toward weight loss. Multiple energy dissipating pathways have been identified in adipose tissue and muscle. The molecular effectors of some of these pathways have been identified, but much is still left to be learned about their regulation. Understanding the molecular underpinnings of metabolic inefficiency in adipose tissue and muscle is required if these pathways are to be therapeutically targeted in the context of obesity and obesity-accelerated diseases.

## Introduction

Adipose tissue is the body’s primary fuel reserve and maintaining a certain threshold has been critical in our evolutionary history for surviving food deprivation and illness.^1^ Obesity is estimated to affect 1 billion people worldwide by 2030,^2^ which imposes a considerable economic burden due to a constellation of obesity-related cardiometabolic complications, such as type 2 diabetes, cardiovascular disease, and cancer. The cause of obesity (accumulation of adipose tissue) is due to positive energy balance, whereby energy intake exceeds energy expenditure. Measurements of free-living energy expenditure using the double-labeled water method have indicated that excess caloric intake is a major driver of weight gain because energy expenditure has remained stable despite a consistent rise in obesity prevalence.^3^ Attempts at promoting negative energy balance via lifestyle-based approaches, such as voluntary food restriction or increased physical activity energy expenditure, prompt physiological counterregulatory mechanisms, such as the constrained total energy theory of metabolism,^4^ that offset the ability to sustain weight loss long term. Furthermore, negative energy balance triggers food intake to re-establish energy balance. However, this latter homeostatic response may be bidirectional, whereby positive energy balance achieved through excess caloric intake triggers satiety^5^ and energy expenditure.^6^ This bidirectionality is consistent with data from humans showing that overfeeding does not result in a similar level of weight gain in all individuals.^7^ Thus, attenuated efficiency in calorie assimilation or differences in energy expenditure may explain the inter-individual variability in weight gain after eating a similar amount of calories, and likely underlie individual differences in susceptibility to obesity in environments where highly palatable, calorically dense foods are copious. For example, experiments in forced overeating have indicated that maintaining elevated body weight that differs from the usual weight is associated with compensatory increases in energy expenditure.^8^ Moreover, overfeeding in identical twins demonstrated that weight gain and fat accrual vary considerably between individuals, with three times more variance among, than within, twin-pairs,^7^ pointing to a genetic component regulating resting energy expenditure or variation in storage of the excess energy in different tissues. Since lifestyle interventions produce a modest degree of weight loss and are typically not sustained long term, other types of anti-obesity interventions are necessary. Pharmacotherapies based on a molecular understanding of appetite regulation have shown astounding (>20%) weight loss.^9^ On the other side of the energy balance equation, low resting and 24-h energy expenditure is predictive of weight gain,^10^ and underweight individuals with a BMI of ∼17 counterintuitively are not leaner than individuals with a BMI of 23 because they exercise more but because they eat less and exhibit a higher than expected resting energy expenditure.^11^ Regardless of the path toward sustained weight loss, maintaining healthy bone and preserving lean mass are paramount to attaining high-quality anti-obesity therapeutics.

Enhancing energy expenditure to combat obesity has been shown in a real-world setting. The proton translocator, 2,4-dinitrophenol (DNP), decreases the thermodynamic backpressure on the mitochondrial electron transport chain by dissipating the electrochemical gradient across the mitochondrial inner membrane. This increases substrate oxidation, respiration, and energy expenditure. In the early 19th century, DNP was widely used as a powerful weight loss drug, but enthusiasm for its use faded due to its narrow therapeutic index.^12^^,^^13^ Despite promising preclinical data over many years, mitochondrial uncouplers have not made it into the clinic, underscoring the safety challenges with this mechanism of energy expenditure induction. In a research setting, achieving long-term negative energy balance by clamping the intake of calories while maintaining an exercise program at 6 days/week, twice per day at 55% VO_2_ max for 100 days was sufficient to induce a mean weight loss of 9%.^14^ Thus, if calories in are clamped, exercise could provide clinically meaningful (5% reduction from baseline)^15^ weight loss in people with moderate overweight/obesity. However, in the real world, the compensatory drive to eat more following exercise, and the behavioral adaptations to increased physical activity, would pose a significant barrier to sustained weight loss. Nevertheless, the DNP studies, the food-clamped exercise studies, and the cross-sectional comparisons of very lean individuals indicate that targeting energy expenditure may be a viable option for promoting negative energy balance and sustaining weight loss. Identifying natural mechanisms within our cells and tissues that promote energy expenditure is thus attractive and has received a lot of attention recently. Key tissues that have been heavily investigated are skeletal muscle and adipose tissue. Energy dissipating pathways that generate heat (thermogenesis) instead of biological work have been identified in both tissues.

Functional redundancy in living systems provides robustness against metabolic and genetic perturbations.^16^ In this way, living systems maintain phenotypic stability in the face of perturbations arising from environmental fluctuations and genetic variation. As detailed in the following section, several mechanisms have been sufficiently demonstrated to promote thermogenesis within adipose tissue and muscle. However, we are far from a complete molecular understanding of how all of these pathways work at a biochemical and biophysical level. Fundamental understanding of the endogenous pathways of energy dissipation and their individual and combined contributions to metabolic rate is essential before the translational potential of energy expenditure can be realized. Moreover, delineating the cell-intrinsic molecular underpinnings of muscle and/or adipose tissue thermogenesis has the potential to uncover novel concepts in metabolic control that may also inform targeting of energy expenditure pathways in disease states.

## Thermogenic adipose tissue

Regulating internal body temperature (thermoregulation) is critical to support normal physiological functions and facilitates the survival and adaptability of endotherms, including human newborns.^17^ Several adipocyte subtypes can dissipate energy into heat. The variation in adipocytes that arise from distinct lineages has been expertly reviewed,^18^^,^^19^ and so will not be the focus here. But it is important to mention that any pathway described herein, while currently having been described using a particular adipocyte subtype as a model system, does not indicate that that pathway is restricted to the cell type in which it has been studied. The quantitative contribution of any single thermogenic pathway (below) among distinct thermogenic adipocyte subtypes, in relation to other thermogenic pathways, is still poorly understood. Adipocytes within brown adipose tissue (BAT) dissipate energy into heat, which is known as non-shivering thermogenesis. These adipocytes are key for thermoregulation in rodents.^20^ Thermoregulation is also supported by shivering thermogenesis and non-shivering thermogenesis in skeletal muscle.^21^^,^^22^^,^^23^^,^^24^ Furthermore, heat conservation mechanisms such as vasoconstriction and insulation are also important for stabilizing body temperature.^25^ Nonetheless, the critical importance of thermogenic fat for homeothermy in rodents is exemplified by the numerous distinct mouse models that lack specific genes controlling adipocyte energy dissipation that cannot stabilize body temperature in a cold environment.^23^^,^^26^^,^^27^^,^^28^^,^^29^ The capacity for thermogenic adipose tissue to dissipate energy and the increasing need to tackle the obesity pandemic forms the basis of attempts at trying to understand the molecular mechanisms driving adipocyte thermogenesis in order to target these pathways to offset obesity-accelerated diseases.

In rodents, thermogenic adipose tissue activity increases in response to excess caloric intake^30^^,^^31^^,^^32^^,^^33^^,^^34^ and provides metabolic protection during nutritional overload.^35^^,^^36^^,^^37^ Although the proportion of thermogenic fat is smaller in humans than in rodents, BAT (a broad term applied to glucose-consuming human thermogenic fat depots) is associated with cardiometabolic health^38^^,^^39^^,^^40^ and promotes energy expenditure in response to feeding^41^ in humans, suggesting a link between thermogenic fat and energy balance. Notably, human thermogenic fat levels tend to be underestimated, as studies have largely quantified it by measuring glucose uptake, even though adipocytes can use other fuel sources (fatty acids, amino acids, and citric acid cycle intermediates) for heat production.^42^^,^^43^ Thermogenic fat in humans and rodents is responsive to reductions in environmental temperature. Evidence from gain- or loss-of-function preclinical models indicates that thermogenic fat disproportionately affects glucose homeostasis relative to body weight.^35^^,^^37^ Similarly, external temperature has a large effect on type 2 diabetes in humans independent of obesity.^44^ Initial prospective clinical studies have shown that increasing the metabolic activity of thermogenic fat is associated with improved insulin sensitivity and cardiometabolic health, even without weight loss.^45^ To exploit adipocyte thermogenesis for therapeutic gain, it is important to decipher the effector proteins and how their activity is regulated, and it will be essential to delineate the quantitative contributions of all energy-dissipating pathways in relation to other thermogenic pathways and whether multiple pathways can be simultaneously activated.

**Figure 1:**
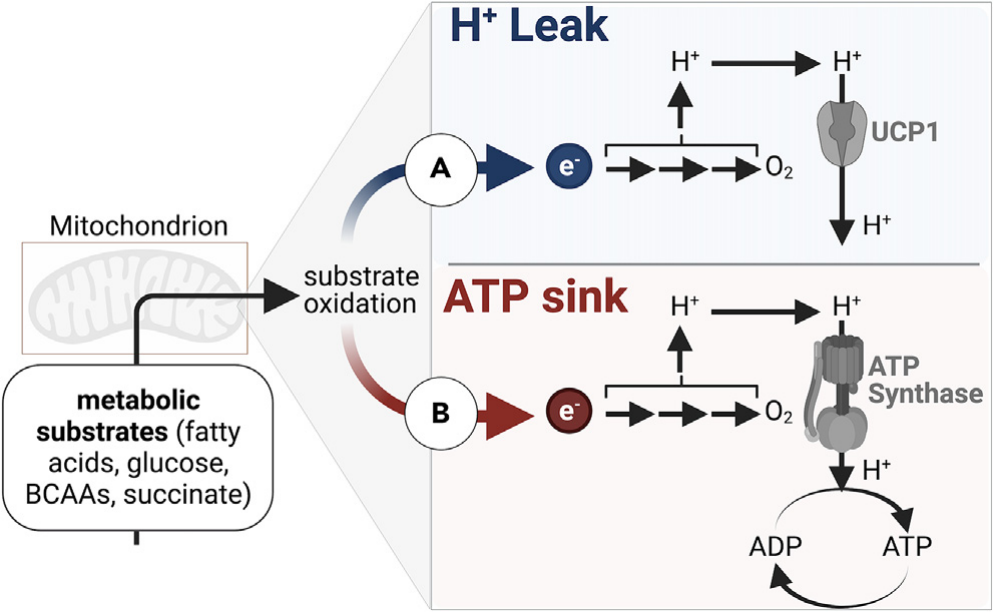
Different modes of thermogenesis Cartoon of thermogenesis by (A), proton re-entry independently of ATP synthesis (H+ leak) or (B), promoting substrate oxidation by ATP turnover (ATP sink).

Brown adipocytes are packed with mitochondria, which are the major site of cellular respiration. The rate of oxygen consumption serves as a measure of oxidation of reducing equivalents donated to the electron transport chain by substrate breakdown. Importantly, macronutrient oxidation is coupled to the synthesis of a fixed number of ATP molecules. This constraint can be bypassed for thermogenesis by disconnecting macronutrient oxidation from ATP synthesis via uncoupling protein 1 (UCP1)^46^ or by increasing ATP turnover.^47^^,^^48^ During the former, UCP1 promotes proton leak from the mitochondrial intermembrane space to the matrix (Figure 1A). Accordingly, mice genetically lacking *Ucp1* in the germline (herein referred to as germline *Ucp1*^−/−^ mice) have been the primary tool for studying thermogenesis. However, thermogenesis can also be promoted by coupling macronutrient oxidation to ATP synthesis, supporting futile cycles that accelerate ATP turnover (Figure 1B).

## Thermogenesis via uncoupling

Upon a thermogenic stimulus, such as cold exposure, free fatty acids (FAs) liberated from stored triacylglycerides (TAGs) or triglyceride-rich lipoproteins in the blood enter mitochondria and activate proton leak via UCP1, a member of the mitochondrial carrier family of transporters that is an integral membrane protein embedded in the mitochondrial inner membrane. The re-entry of protons from the mitochondrial intermembrane space to the matrix stimulates substrate oxidation, oxygen consumption, and thermogenesis. Readers are referred to expert reviews on UCP1-dependent thermogenesis.^49^^,^^50^^,^^51^ Somewhat interestingly, the most unexpected phenotype of germline *Ucp1*^−/−^ mice is their normal resting energy expenditure and resistance to weight gain on a high-fat diet under standard room temperature housing conditions (∼20°C–24°C; assumed temperature unless otherwise noted).^26^^,^^52^ This was an unforeseen observation given the involvement of BAT in diet-induced thermogenesis,^30^ and the idea that UCP1 was the only key thermogenic effector. Because germline *Ucp1*^−/−^ mice triggered compensatory thermoregulatory mechanisms that were not as efficient in distributing heat throughout the body, this may have necessitated burning of more fuel than would otherwise be needed if UCP1 were active.^53^ Soon after, it was proposed that thermoneutral housing was required to reveal the effect of UCP1 on mitigating obesity;^31^ however, independent studies have now demonstrated that obesity is not potentiated in germline *Ucp1*^−/−^ mice at thermoneutrality.^52^^,^^54^^,^^55^^,^^56^^,^^57^^,^^58^ About 65% of the weight loss that occurs from β_3_ adrenergic receptor (ADRB3) stimulation does not require UCP1, while the remaining 35% is UCP1-dependent.^58^ Decades of work have verified a role for UCP1 in adipocyte thermogenesis. However, current research efforts also clearly indicate that UCP1-independent thermogenic pathways exist and that their activation explains, at least partly, the paradoxical energy-dissipating physiological outputs from a diverse set of *in vitro* and *in vivo* studies. Tapping into energy dissipation for therapeutic intervention necessitates a clear understanding of all thermogenic pathways.

## ATP synthesis during adipocyte thermogenesis

Olov Lindberg indicated that thermogenic adipocytes might use a combination of ATP turnover and uncoupling to support thermogenesis.^59^ However, the severe thermogenic and respiratory impairment of germline *Ucp1*^−/−^ mice has led to the conventional view that UCP1 is the sole effector protein of non-shivering thermogenesis in adipocytes.^23^ But it is important to note that brown adipocytes of germline *Ucp1*^−/−^ mice acquire secondary changes to critical metabolic circuits (reduced electron transport chain abundance, impaired cristae morphology, and potentially other defects) that are required to trigger and sustain thermogenesis by any mechanism. These changes may confound the ability to resolve the physiological relevance of adipocyte-intrinsic noncanonical thermogenic mechanisms that also rely on these central metabolic components. In recent years, the idea that thermogenesis occurs through futile cycles that accelerate ATP turnover has gained attention.^48^^,^^60^^,^^61^^,^^62^^,^^63^^,^^64^^,^^65^^,^^66^ BAT harvested during physiological activation of thermogenesis (cold exposure) indicates that thermogenic adipocytes maintain a high phosphorylation potential,^67^ and that the calculated adenylate energy charge^68^ is maintained around 0.8, which is within the normal physiological range displayed by a wide variety of eukaryotes and prokaryotes.^69^ These data indicate that ATP-producing reactions are in balance with major ATP-consuming reactions during adipocyte thermogenesis. When mitochondria from thermogenic fat are purified and respired under nonphysiological conditions (without purine nucleotides), the mitochondrial electrochemical gradient is dissipated via UCP1.^49^ However, this is not necessarily the case during activation of UCP1-dependent thermogenesis in intact cells using physiologically relevant levels of adrenergic agonists (discussed in the following section). Also, concentrations of purine nucleoside di- and triphosphates in the cytosol of mammalian cells are thought to be in the range well above those required to completely inhibit proton leak in isolated mitochondria.^49^^,^^70^ Thus, during physiological activation of UCP1, lipolysis-mediated FA liberation must overcome purine nucleotide inhibition on UCP1 and must increase beyond their removal by long-chain acyl CoA synthetase (ACSL, of which there are 5 isoforms, makes acyl-CoAs that do not activate UCP1) and beta oxidation.^49^ Therefore, the loose coupling of adipocyte mitochondria during thermogenesis is not an all-or-none response but, as reported decades ago, is one that is graded in nature.^67^ If the phosphorylation potential of BAT during physiological activation of thermogenesis maintains a normal adenylate energy charge, and if the response to adrenergic stimulation is graded, one would expect that the coupled and uncoupled contributions to thermogenesis would, for example, vary depending on the particular strength of the adrenergic signal. Thus, it is certainly reasonable that uncoupled and coupled respiration could occur in the same cell during thermogenesis. However, it is also possible that unique cell types will favor one mechanism over the other. Importantly, the amount of adrenergic agonist that mimics the physiological thermogenic state experienced by cold-exposed BAT should be, but is rarely, considered when modeling thermogenesis in reductionist (isolated mitochondria or cell) systems. Modeling thermogenesis in acutely isolated mature brown adipocytes with levels of noradrenaline that more closely approximate the level released during cold (albeit still supraphysiological) has revealed that 30%–40% of noradrenaline-driven thermogenesis is ATP coupled.^65^^,^^71^ Lower doses of noradrenaline, to physiological levels, may tip the balance even further toward thermogenesis by ATP-coupled respiration.

The capacity for a proton translocator to stimulate respiration by collapsing the electrochemical gradient is indicative of how much of the gradient can be released by the uncoupler, but it cannot diagnose the proportion of noradrenaline-stimulated thermogenesis that is coupled to ATP synthesis because the respiratory chain cannot distinguish between dissipative pathways that increase proton conductance directly or through ATP synthesis and turnover.^72^ In this respect, agents that inhibit ATP synthesis are a better diagnostic tool to determine the degree of thermogenesis that is coupled to phosphorylation. The inhibitory action of oligomycin on stimulated respiration of intact brown adipocytes^65^^,^^71^^,^^73^ confirms the presence of an oligomycin-sensitive ATPase in BAT mitochondria, which is consistent with loosely coupled mitochondria being capable of oxidative phosphorylation during thermogenesis. The quantitative contributions of UCP1 versus ATP-consuming futile cycles among distinct thermogenic cell types and their individual and combined physiological relevance *in vivo* are still a wide open question, but have begun to be investigated. For the remainder of this perspective, I will focus on energy expenditure via ATP turnover in adipose tissue and muscle.

## Futile creatine cycle

**Figure 2:**
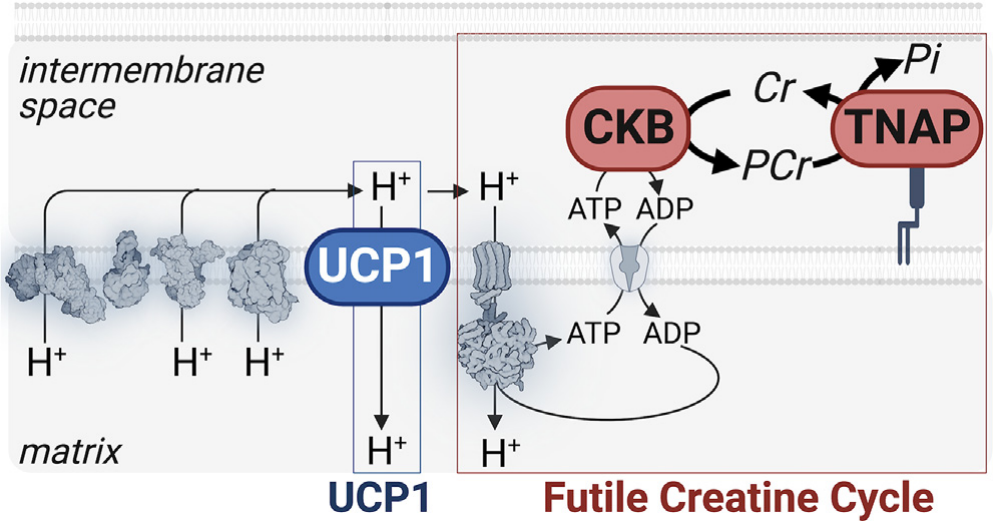
Thermogenesis by uncoupling protein 1 (UCP1) and the futile creatine cycle Complexes I, III, and IV of the electron transport chain extrude protons (H^+^) from the matrix to the intermembrane space to generate an electrochemical gradient across the mitochondrial inner membrane. Thermogenesis mediated by proton (H^+^) leak via UCP1 or by ATP turnover via the futile creatine cycle occurs because of H^+^ re-entry back to the matrix which relieves the thermodynamic backpressure on the electron transport chain, which promotes substrate oxidation, respiration, and thermogenesis. CKB, Creatine kinase B; TNAP, Tissue non-specific alkaline phosphatase, UCP1, uncoupling protein 1, Cr, creatine; PCr, phosphocreatine; Pi, inorganic phosphate.

A role for creatine in UCP1-independent thermogenesis *in vivo* is supported by work from independent groups using multiple mouse lines.^34^^,^^48^^,^^65^^,^^66^^,^^74^^,^^75^^,^^76^^,^^77^ The current working model of creatine-mediated thermogenesis is the futile creatine cycle (Figure 2). This model posits that creatine undergoes a phosphorylation/de-phosphorylation cycle, which is fueled by ATP synthesis.^47^^,^^48^ This framework has been critical for the elucidation of two key effector proteins localized to mitochondria in thermogenic adipocytes that are required for this UCP1-independent thermogenic pathway in mice: creatine kinase B (CKB) and tissue non-specific alkaline phosphatase (TNAP)^48^^,^^65^^,^^66^ (Figure 2). Notably, the model of the futile creatine cycle predicted that a phosphocreatine hydrolytic enzyme, localized to the mitochondria, would exist in thermogenic fat; the identification of TNAP as a protein that fulfilled these criteria was a direct consequence of the futile creatine cycle model. In mice genetically lacking either *Ckb* or *Alpl* (the gene encoding TNAP protein), weight (primarily fat mass) gain is elevated on a high-fat diet compared to littermate controls. Moreover, loss of adipocyte *Ckb* results in poorer glucose tolerance after high-fat diet feeding in an adiposity-independent manner, again pointing to a disproportionate influence of thermogenic fat over glucose homeostasis relative to body weight. Genetic or pharmacological depletion of CKB or TNAP, respectively, impaired noradrenaline-stimulated thermogenesis, in a brown adipocyte-intrinsic manner by about 40%, arguing for direct effects on these cells.^65^ Furthermore, inhibition of TNAP activity did not decrease noradrenaline-stimulated respiration in *Ckb*-null brown adipocytes, indicating that CKB and TNAP function within the same pathway, which is consistent with the futile creatine cycle model. The requirement of CKB and TNAP for noradrenaline-stimulated thermogenesis was also dependent on ATP synthase, another prediction of the model. Using the same dose of noradrenaline as previously described, Nedergaard and Lindberg demonstrated that thermogenesis was ATP linked, but instead of oligomycin they used atractyloside.^71^ These independent studies used brown adipocytes with intact *Ucp1*, indicating that when thermogenesis is modeled using noradrenaline concentrations that more closely mimic the concentrations found *in vivo* during physiological activation of thermogenesis,^78^^,^^79^^,^^80^^,^^81^^,^^82^^,^^83^ both uncoupling and ATP turnover are simultaneously triggered. Understanding the relative contribution of multiple thermogenic pathways will necessitate modeling the triggers of thermogenesis at physiological concentrations. It is likely that the relative contribution of each individual pathway will be cell and context dependent.

Therapeutic targeting of adipocyte thermogenesis will require the effects to be sustained long enough to meaningfully impact nutrient utilization. ADRB3 agonists or noradrenaline have been the preferred approaches to examine adipocyte thermogenesis in preclinical models. And there is currently fervent debate on whether human BAT thermogenesis is stimulated better with ADRB2 and ADRB3 agonists.^62^^,^^84^ Importantly, Gα_s_-coupled cAMP production through GPR3 demonstrated large increases in energy expenditure *in vivo*, associated with inductions of *Ucp1*^85^ and *Ckb* and *Alpl*,^65^ indicating that GPR3 can coordinate both UCP1-dependent and -independent thermogenesis. While Gα_s_-coupled cAMP production has dominated the focus of adrenergic stimulation of adipocyte-mediated energy dissipation,^62^^,^^84^^,^^85^ it has long been appreciated that noradrenaline engages G-protein-coupled receptors (GPCRs) aside from βARs on brown adipocytes.^86^^,^^87^ For example, αAR signaling in rodent and human adipocyte metabolism has been demonstrated^87^^,^^88^^,^^89^^,^^90^; yet, the particular αAR subtype, the class of G protein that it is coupled to, and the effector protein(s) that transduce αAR signaling into a thermogenic output have only recently been discerned, wherein ADRA1A was identified to be the αAR subtype that is sufficient to potentiate adipocyte thermogenesis through physical and functional coupling to Gα_q_ in mouse adipocytes. In addition, ADRA1A-Gα_q_ signaling was shown to trigger adipocyte thermogenesis, at least partially, through effector proteins implicated in the futile creatine cycle, which is consistent with thermogenesis downstream of αAR activation being ATP linked.^65^^,^^91^

Using designer receptors exclusively activated by designer drugs-based chemogenetics, mice capable of activating Gα_q_ signaling selectively in adipocytes have been generated.^65^ Critically, a sustained and adaptive elevation of whole-body energy expenditure was reached through combined adipocyte-selective Gα_q_ and Gα_s_ signaling. In contrast, the adaptive thermogenic output elicited by dual Gα_s_ and Gα_q_ signaling was absent in mice genetically lacking *Ckb* in adipocytes. With respect to whole-body energy expenditure, ADRB3 signaling alone was not adaptive. Thus, Gα_q_ signaling within mature adipocytes potentiates the stimulation of energy expenditure elicited by Gα_s_ signaling in a manner that is genetically dependent on adipocyte *Ckb*, and the sustained and cumulative rise in energy expenditure through combined Gα_s_ and Gα_q_ activation is CKB-dependent. The generation of these new genetic tools has highlighted some limitations to exploring adipocyte thermogenesis *in vivo* via selective ADBR3 agonism or noradrenaline injections. The former does not take into account the key role that other receptors, such as ADRA1A, play in thermogenesis, and the latter is not adipocyte-selective. Future work should examine whether the level of sustained energy expenditure that is elicited by combined Gα_q_ and Gα_s_ signaling is sufficient to improve systemic metabolic parameters in the context of obesity-accelerated glucose intolerance, insulin resistance, tissue inflammation, and fibrosis. Whether the capacity for Gα_q_ signaling to promote adipocyte thermogenesis is unique to its potentiating effects on the ADRB3-Gα_s_ axis or is more broadly applicable to other Gα_s_-stimulating factors also merits further investigation. Finally, examining the contribution of UCP1 to the profound thermogenic effects of combined Gα_q_ and Gα_s_ signaling *in vivo* should be explored.

The transcriptional control of UCP1-independent thermogenesis is poorly researched, but recently the transcriptional regulation of effectors of the futile creatine cycle has begun to be delineated. Fat-selective ablation of EBF1/2, ERRα/γ, or PGC1α inhibited cold-induced CKB induction, and cold-stimulated TNAP expression was dependent on EBF1/2 and PGC1α.^65^ Since EBF1/2, ERRα/γ, and PGC1α have all been demonstrated to regulate UCP1, their control over CKB and TNAP provide compelling support for the idea that distinct thermogenic pathways can run in parallel. PR domain-containing 16 (PRDM16) is a key regulator of thermogenic adipocyte determination and differentiation.^92^ Genetic depletion of the CUL2-APPBP2 ubiquitin E3 ligase promoted thermogenic activation and PRDM16 stabilization.^93^ Interestingly, while loss of *Appbp2* promoted PRDM16-dependent *Ucp1* induction, *Ckb* mRNA levels were elevated by *Appbp2* loss in a PRDM16-independent manner.^93^ These data suggest that APPBP2 may support the degradation of thermogenic activators that extend beyond PRDM16, which are required for cold-induced *Ckb* mRNA and protein induction.^65^ Mice with adipocytes that genetically lack zinc-finger protein 423 (ZFP423), a transcriptional repressor of EBF2 and thus the thermogenic gene program,^94^ exhibit increased adipocyte thermogenesis and CKB protein expression in subcutaneous fat, and protection from obesity during mistimed feeding.^34^ The transcriptional regulation of other UCP1-independent thermogenic pathways within adipocytes, such as the futile calcium cycle and futile lipid cycle (detailed in the following section), is ripe for investigation.

## Futile calcium cycle

**Figure 3:**
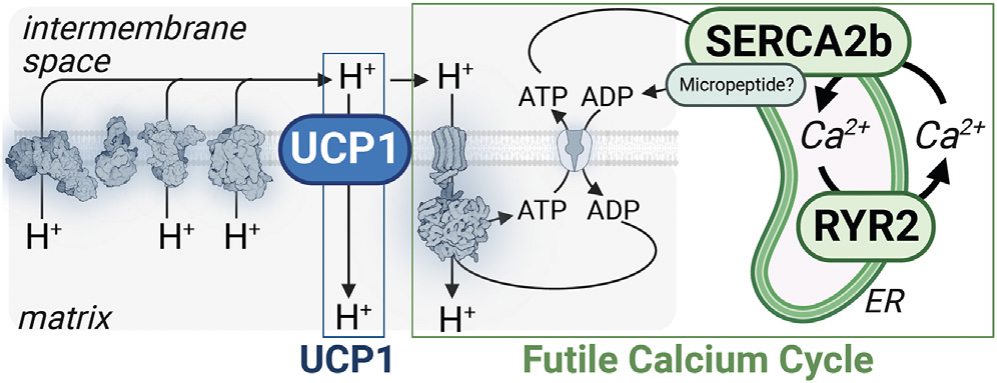
Thermogenesis by UCP1 and the futile calcium cycle Thermogenesis mediated by proton (H^+^) leak via UCP1 or by ATP turnover via the futile calcium cycle occurs because of H^+^ re-entry back to the matrix which relieves the thermodynamic backpressure on the electron transport chain, which promotes substrate oxidation, respiration, and thermogenesis. ER, endoplasmic reticulum; SERCA2b, sarcoplasmic and endoplasmic reticulum calcium ATPase 2b; RYR2, Ryanodine receptor 2, UCP1, uncoupling protein 1, Ca^2+^, calcium.

The major storage site of intracellular calcium (Ca^2+^) is in the sarcoplasmic reticulum (SR) of striated muscle and the endoplasmic reticulum in other cell types. Ryanodine receptors (RyRs) are major Ca^2+^ release channels located along the SR/endoplasmic reticulum. Cytosolic Ca^2+^ abundance is regulated by RyR-mediated Ca^2+^ release into the cytosol and subsequent uptake of Ca^2+^ by the sarco/endoplasmic reticulum Ca^2+^ ATPase (SERCA) pump. Billfish, such as marlins, sailfish, spearfish, and swordfish, have extraocular muscles consisting of cells specialized for heat production, not contraction, wherein energy from ATP hydrolysis is used to pump Ca^2+^ into the SR, followed by calcium release from the SR by physiologically unique isoforms of the Ca^2+^ ATPase (SERCA1B) and RyR1-slow, respectively.^95^^,^^96^ This constitutes a futile calcium cycle and thermogenesis. In adipose tissue, Fabp4 promoter-controlled PRDM16 overexpression can rescue the acute cold intolerance of germline *Ucp1*^−/−^ mice.^63^ This effect was tied to futile calcium cycling within *Ucp1*^−/−^ subcutaneous adipocytes, in a cycle catalyzed by SERCA2b and Ryr2^63^ (Figure 3). Recent work (detailed in the following section) has now shown that elevating Ca^2+^ influx into subcutaneous adipocytes increases thermogenesis in both wild-type and *Ucp1*^−/−^ adipocytes, and is genetically dependent on *Atp2a2* (gene encoding SERCA2).^97^ This work also highlighted a key role played by αAR signaling in thermogenesis.^63^ This SERCA2b-RYR2 axis was shown to be important in germline *Ucp1*^−/−^ beige adipocytes, but dispensable in brown adipocytes.

Adipocyte-selective expression of light-gated channelrhodopsin-2 (ChR2) has been employed to trigger Ca^2+^ influx into adipocytes via optogenetics, which was sufficient to protect mice from diet-induced obesity, even in the presence of intact *Ucp1*.^97^ Interestingly, triggering Ca^2+^ influx was sufficient to enhance beige adipocyte respiration.^97^ This is distinct from mature brown adipocytes, where Ca^2+^ signals from selective αAR signaling require a Gα_s_ stimulus,^65^ presumably to release the fuel, in order to potentiate the thermogenic response. The ability for cultured beige adipocytes to only require Ca^2+^ influx may be due to a higher basal tone of lipolysis in this model system or perhaps a reliance on alternative fuels (glucose oxidation was elevated upon ChR2 stimulation^97^) that are present in the cellular culture media. Whatever the cause, this is certainly an interesting mechanistic avenue to pursue. Finally, an important advance will clearly lie in understanding the acute trigger of futile calcium cycling. In skeletal muscle, micropeptides such as sarcolipin and neuronatin (detailed in the following section) have been shown to uncouple SERCA activity and thus thermogenesis via futile calcium cycling. Deciphering whether beige adipocytes express a unique micropeptide that uncouples SERCA2b-mediated ATP hydrolysis will be an important advance. Since PRDM16 regulates a wide variety of genes related to brown and beige fat cell determination and differentiation,^92^^,^^98^ delineating whether PRDM16 gain of function normalizes body temperature of germline *Ucp1*^−/−^ mice is tied to restored cristae morphology and electron transport chain abundance would be an interesting avenue of pursuit.

## Futile lipid cycle

**Figure 4:**
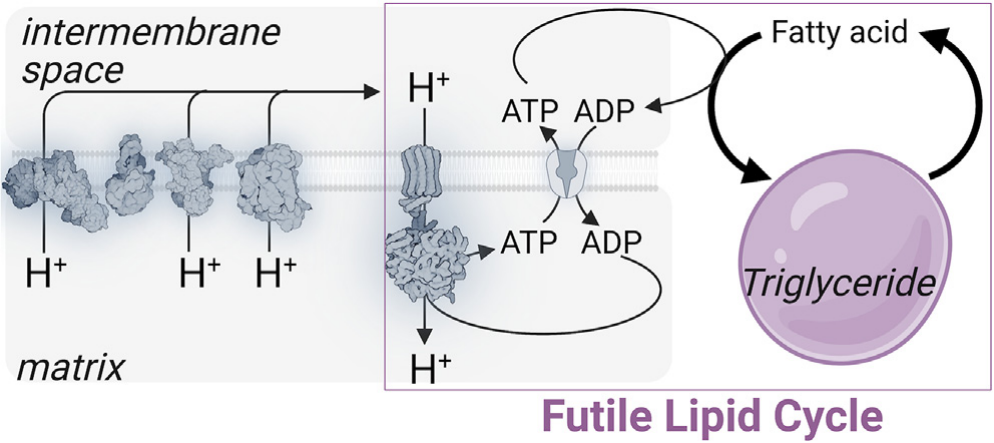
Thermogenesis futile lipid cycle Thermogenesis mediated by ATP turnover via the futile lipid cycle occurs because of H^+^ re-entry back to the matrix which relieves the thermodynamic backpressure on the electron transport chain, which promotes substrate oxidation, respiration, and thermogenesis. This cycle has been reported to occur primarily in white adipocytes, but has been shown to also occur in brown adipocytes that genetically lack *Ucp1* or where the mitochondrial pyruvate carrier has been pharmacologically inhibited.

Ball and Jungas put forward the hypothesis that adipose tissue produces heat by the exothermic cycle of triglyceride hydrolysis and re-esterification as a result of their studies in white adipose tissue.^99^ In this cycle, lipolysis of stored TAGs into FAs and glycerol, or monoacylglyceride (MAG), or diacylglyceride (DAG) intermediates, are followed by the re-esterification of activated FAs back into MAGs, DAGs, or TAGs. The re-esterification pathway requires the presence of glycerophosphate, which is acquired from ATP investment from glucose metabolism or glycerol kinase activity, and the ATP-consuming process of FA activation into FA-CoA. Since the re-esterification arm of this cycle uses ATP, this process dissipates energy and drives thermogenesis (Figure 4). Biochemical studies on BAT *in vitro* could not detect substantial lipid cycling.^100^ More recently, inhibition of enzymes involved in re-esterification did not reduce thermogenesis in wild-type brown adipocytes *in vitro.*^101^ Furthermore, the molar ratio of released FAs/glycerol during stimulation of brown adipocytes by noradrenaline was approximately 2.5, a value that is sufficiently close to 3 to indicate that there was not much lipid cycling under these conditions.^73^ Since glycerol release can also arise from glucose metabolism,^102^ the idea that glycerol release is a quantitative marker of TAG hydrolysis has been questioned,^103^ and so glycerol release may overestimate the degree of lipolysis. Recently, brown adipocytes have been reported to undergo futile lipid cycling *in vitro*, following genetic ablation of *Ucp1* or inhibition of the mitochondrial pyruvate carrier (MPC).^61^^,^^104^ Upon *Ucp1* ablation, lipid cycling was reported to be activated downstream of adrenergic stimulation,^61^ while MPC inhibition was reported to trigger a lipid subcycle independently of adrenergic stimulation and in a diglyceride acyltransferase (DGAT)-independent manner.^104^ DGAT catalyzes the formation of TAGs from DAGs and FA-CoA.^105^ Conversely, mice with *Mpc* ablation in thermogenic fat did not display altered whole-body energy expenditure, yet they were cold sensitive.^106^ These *in vivo* data indicate that complete glucose oxidation is required to stabilize body temperature in the cold, and that if futile lipid cycling is activated upon genetic ablation of *Mpc in vivo*, as it is with pharmacological blockade *in vitro*, it cannot compensate to stabilize body temperature in the cold. In humans, by taking into account cold-stimulated glycerol release and beta oxidation, futile lipid cycling has been suggested to contribute approximately 30% of the cold-mediated rise in energy expenditure.^62^ Certainly, if futile lipid cycling is primarily restricted to white adipocytes, this could be an interesting therapeutic angle to pursue in humans with copious amounts of white adipocytes. Although, it is interesting to contemplate whether loss of stored TAGs through futile lipid cycling would eventually prohibit further activation of this cycle. Perhaps, specific high fat dietary interventions would need to be adopted to maintain the activation of this pathway after sufficient weight loss was achieved.

The presence of a potent glycerophosphate dehydrogenase in brown fat^107^ will limit the rate of FA re-esterification through alpha-glycerophosphate removal. However, glycerophosphate dehydrogenase activity is lower in white adipose tissue, which may render white adipocytes better equipped to re-esterify FAs. As noted previously, thermogenesis via ATP-consuming FA re-esterification could arise from TAG hydrolysis to FAs and glycerol and then re-esterification back to TAGs. Alternatively (although not mutually exclusive), subcycles between TAGs and FAs and glycerol could exist. Whether such a subcycle exists that is under adrenergic control and whether it contributes to physiologically relevant amounts of thermogenesis remains to be seen, although the DGAT-dependent step seems to have been ruled out.^101^^,^^104^ Recently, tracing based on alkyne-labeled FAs with click chemistry and mass spectrometry has been used to provide direct detection of futile lipid cycling in white and brown adipocytes *in vitro.*^103^ Interestingly, futile lipid cycling was detected in the absence of an extrinsic thermogenic stimulus, providing further evidence that lipid cycling has some capacity to operate basally. In addition, beta oxidation was shown to compete for the FA pool that is liberated from stored TAGs, indicating that, like glycerophosphate dehydrogenase activity, FA oxidation will compete with futile lipid cycling. This raises some interesting mechanistic questions. For example, what metabolic fuels would be required to sustain futile lipid cycling? How would reducing equivalents derived from FA oxidation, that support mitochondrial ATP synthesis, impact the futile lipid cycle directly (via competition noted previously) or indirectly by supporting other ATP-consuming futile (creatine or calcium) cycles? Some additional unanswered areas ripe for investigation will be to (1) determine whether genetic modulation of predicted effector proteins of futile lipid cycling contribute to physiologically meaningful amounts of energy dissipation *in vivo*; (2) decipher the quantitative contribution of various possible short (TAG/DAG cycling) and long (TAG/MAG, TAG/glycerol, DAG/glycerol, MAG/glycerol cycling), with the caveat however of the appeared dispensability of the DGAT-catalyzed step of DAG to TAG; and (3) uncover how acute control over lipid cycling is regulated by extrinsic thermogenic signals.

## Non-shivering thermogenesis in muscle

**Figure 5:**
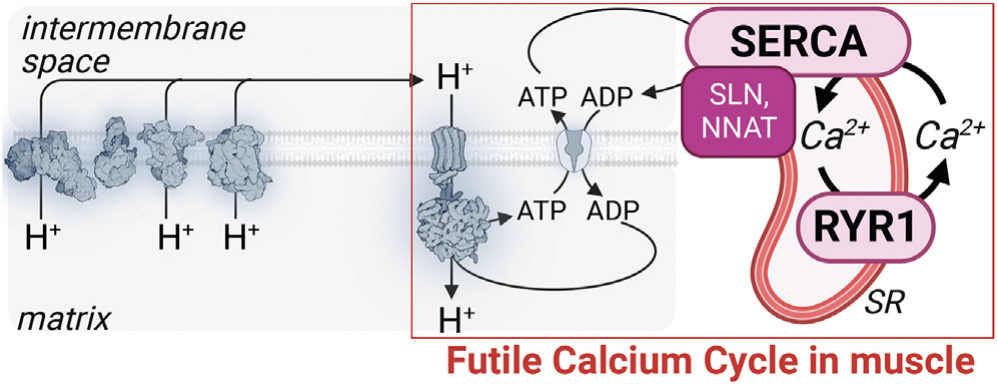
Thermogenesis by futile calcium cycling in muscle Thermogenesis mediated by ATP turnover via muscle-based futile calcium cycling occurs because of H^+^ re-entry back to the matrix which relieves the thermodynamic backpressure on the electron transport chain, which promotes substrate oxidation, respiration, and thermogenesis. SR, sarcoplasmic reticulum; SERCA, sarcoplasmic and endoplasmic reticulum calcium ATPase; RYR1, Ryanodine receptor 1; SLN, Sarcolipin; NNAT, Neuronatin; Ca^2+^, calcium.

Heat generation via shivering and non-shivering thermogenesis in skeletal muscle are important thermoregulatory mechanisms.^21^^,^^22^^,^^23^^,^^24^ Shivering involves rapid muscle contraction and relaxation and is dependent on intracellular Ca^2+^. SERCA harnesses energy from ATP to pump Ca^2+^ into the SR lumen in a 2:1 stoichiometry of Ca^2+^:ATP. Sarcolipin (SLN, encoded by the *Sln* gene) is a single-pass transmembrane peptide that interacts with SERCA to decrease the Ca^2+^:ATP stoichiometry, which accelerates ATP turnover. In combination with RYR1-mediated Ca^2+^ leak, there is enhanced substrate oxidation and ADP-dependent respiration to produce ATP to support this thermogenic process^108^ (Figure 5). Mice lacking *Sln* in the germline gain more weight than control mice during high-fat diet feeding,^21^ while skeletal muscle-selective *Sln* overexpression provides protection from high-fat diet-induced obesity.^109^ Recently, neuronatin (NNAT) has been shown to also impair ATP-mediated Ca^2+^ pumping by SERCA,^110^ and whole-body *Nnat* knockout mice display reduced energy expenditure.^111^ Unlike SLN, NNAT expression is not restricted to skeletal muscle, and so the involvement of NNAT in cellular thermogenesis within muscle and beyond merits further investigation, particularly using models where *Nnat* is ablated in a cell-type-selective manner.

Skeletal muscle myosin ATPase activity has also been proposed to contribute to heat production in relaxed skeletal muscle upon transition from the super relaxed state (with highly inhibited ATPase activity) to the conventional relaxed state,^112^ a transition that is linked to structural remodeling of the thick filament.^113^ These two states are distinct from the high ATP turnover rate of active muscle, therefore, indicating that contractile proteins may contribute to skeletal muscle non-shivering thermogenesis. Simply by representing a large fraction of body mass, skeletal muscle non-shivering thermogenesis represents a viable therapeutic target for driving energy expenditure to offset obesity. Of course, the degree of stimulated thermogenesis by muscle would have to be tuned in such a way as to not interfere with its contractile function. Additional details of muscle non-shivering thermogenesis have been expertly reviewed.^114^^,^^115^

## Futile RNA cycle

Another energy-dissipating pathway, known as the futile RNA cycle, has been studied in mice genetically lacking *Maf1* from the germline (*Maf1*^−/−^).^116^^,^^117^ MAF1 is a negative regulator of RNA polymerase (Pol) III transcription. *Maf1*^−/−^ mice are resistant to diet-induced obesity with several proposed contributing mechanisms such as reduced food intake and possibly increased energy expenditure.^117^ Notably, *Maf1*^−/−^ mice exhibit lower body weight compared with age-matched controls after weaning.^117^ At the molecular level, energy dissipation in *Maf1*^−/−^ mice was posited to result from sustained synthesis and degradation of Pol III transcripts, which was associated with elevated nucleotide synthesis to support the tRNA production arm of this cycle.^116^ Future work should focus on experiments in mice of the same weight to confirm whether the elevation of energy expenditure precedes weight loss in *Maf1*^−/−^ mice. Furthermore, experiments at thermoneutrality may attenuate the superimposing effect of standard room temperature housing on metabolic heat generation that might occur in a mouse with growth defects. Lastly, tissue-specific knockouts are worthy of pursuit in order to define the key tissues that, when running the futile RNA cycle, contribute most toward whole-body energy expenditure.

## Conclusion and future perspectives

A key goal of obesity research is to understand the cellular mechanisms underlying energy dissipation in physiological and disease states. To do so, it is important to model the physiological state in reductionist systems and to generate genetic models where thermogenic signals/outputs are perturbed in a cell-type-selective manner *in vivo*. The infusion of noradrenaline has been an important model system to monitor thermogenesis *in vivo.*^118^ However, an important caveat of this approach is that noradrenaline will signal through non-adipose mechanisms. This was elegantly demonstrated where the thermogenic response to noradrenaline, caused by cold acclimation, was maintained despite surgical removal of the interscapular BAT.^118^ Additionally important is that measurements based on noradrenaline injections are constrained to measurements around 1 h or less in animals that are typically under anesthesia. Therefore, this approach is not ideal for monitoring long-term and sustained effects of effectors on thermogenic output. Another approach typically used is administration of the ADRB3-selective agonist CL316,243. This approach is quite selective to adipocytes in preclinical mouse models, yet only activates the Gα_s_ arm of GPCR signaling, thus not allowing for the study of parallel signaling events that are critical potentiators of the thermogenic response. An approach more recently developed is via selective peripheral targeting of the modified muscarinic receptor (hM3Dq) to adipocytes and to combine activation of Gα_s_ and Gα_q_ signaling *in vivo*.^65^ This approach can mimic some aspects of noradrenaline (the physiological regulator of thermogenesis), but is advantageous because it is fat selective and can trigger a sustained and adaptive enhancement of thermogenic output *in vivo* that can be measured for weeks.

A comprehensive understanding of the heat-producing pathways within muscle and adipose tissues may offer the possibility of a pharmacotherapy that targets the energy expenditure side of the energy balance equation that is both safe and effective. Although the amount of thermogenic adipose tissue is smaller in humans than in rodents, this does not call into question fundamental work on cell-intrinsic mechanisms of adipocyte thermogenesis, which could be applied to larger adipose depots in humans. It also does not negate the fact that using thermogenic adipocytes as a research tool has the capacity to discover novel concepts in metabolic control that may be applicable to other cell types. The maximum detectable thermogenic fat mass in humans appears to be approximately 1 kg, and in adults 20 to 50 years of age, it ranges from 50 to 500 g, or 0.1% to 0.5% of total body mass.^42^ However, it is also fair to point out that the amount of human thermogenic fat and its influence on total energy expenditure may be underestimated because the field still largely relies on quantifying thermogenic adipose tissue volume using glucose uptake measurements with 18F-fluoro-deoxyglucose-positron emission tomography/computed tomography (18FDG-PET/CT), even though adipocytes use other fuel sources for heat production.^42^^,^^43^ Based on the low total body thermogenic fat volume quantitated by ^18^FDG PET, human thermogenic adipose tissue accounts for <1% of circulating glucose uptake during acute cold exposure. Novel imaging methods may reveal that thermogenic fat displays a higher fractional uptake of other metabolic fuels. State-of-the-art approaches and limitations to human thermogenic adipose tissue research have been expertly reviewed.^43^

How much energy expenditure is required to attain clinical relevance will likely depend on the outcome measure and target population. To sway whole-body energy balance, activating macronutrient oxidation via energy dissipative mechanisms in muscle or adipose tissue would likely require coupling with an appetite suppressant to impede compensatory elevations in food intake. As thermogenic pathways and determination factors are identified, there is no reason to assume that the activity or amount of thermogenic adipose tissue cannot be altered. The characterization of adipocyte heterogeneity and control mechanisms of thermogenic adipocyte programming is critical for the development of future therapeutic avenues to exploit the unique thermogenic properties of adipocytes. New therapeutic treatments for metabolic diseases surrounding adipocyte dysfunction may benefit from a refined understanding of the diversity in biochemical pathways contributing to thermogenic respiration. If catabolic activity of adipocytes can be increased without side effects, there may still be a place for targeting human adipose tissue in treating obesity and obesity-associated disease. Most importantly, adipocyte-driven energy dissipation does not need to be limited to adipocytes derived from classical BAT. Brown adipocytes can be used as a model system to harness our understanding of thermogenic mechanisms that could then be applied to white adipose depots, which no one would deny are plentiful in humans. Modulating energy balance to maximize cardiometabolic health can benefit from an understanding of the counterregulatory mechanisms that decrease energy expenditure upon food intake suppression and conversely stimulate food intake upon enhanced energy expenditure. A combination of strategies may find a sweet spot for optimal healthy weight loss with minimal side effects.

The next phase for the field studying adipocyte thermogenesis will be to understand how, at a biophysical level, these remarkable and heterogeneous set of cells can generate heat independently of UCP1, and what other roles thermogenic adipocytes play beyond thermogenesis. The main adipocyte-mediated UCP1-independent thermogenic pathways, currently under investigation, display some similarities and some differences. The futile creatine cycle occurs in various thermogenic (beige and brown) adipocytes. In contrast, calcium cycling appears to be restricted to beige fat. Lipid cycling occurs in wild-type white adipocytes, while to detect it in brown fat *in vitro* requires germline *Ucp1* ablation or pharmacological perturbation of glucose metabolism. Delineating these distinctions and nuances may help decipher governing mechanisms that trigger a combination of these pathways that could have physiologically relevant and clinically meaningful implications. Regarding muscle non-shivering thermogenesis, a key regulator is SLN. However, NNAT also may play an important role. How SLN- and NNAT-mediated non-shivering thermogenesis in skeletal muscle are coordinated will be an interesting area of future research.
